# Rectal Dieulafoy Lesion Managed by Hemostatic Clips

**DOI:** 10.4021/jocmr945w

**Published:** 2012-11-11

**Authors:** Hyung Hun Kim, Joo Hoon Kim, Sung Eun Kim, Seun Ja Park, Moo In Park, Won Moon

**Affiliations:** a Department of Internal Medicine, Kosin University College of Medicine, Busan, Korea

**Keywords:** Hematochezia, Hemostasis, Colonoscopy, Clip

## Abstract

The classic Dieulafoy lesion is a minute gastric mucosal defect which bleeds massively from an exposed artery. The typical endoscopic appearance of this lesion is a single, round mucosal defect with an artery protruding from its base in the absence of surrounding ulceration. We encountered an 89-year-old man who developed sudden massive fresh rectal bleeding. The source of hemorrhage was found at colonoscopy after careful irrigation and inspection to be a Dieulafoy lesion situated in rectum. Hemostasis was achieved successfully with epinephrine injection and endoscopic hemostatic clipping.

## Introduction

Dieulafoy lesion is an uncommon but well-recognized cause of significant gastrointestinal bleeding. Initially described by Gallard in 1884, Dieulafoy was the first to characterize this lesion in 1898 when he described three cases presenting with massive upper gastrointestinal bleeding [1]. Although usually found in the stomach, Dieulafoy lesions have been described in the rest of the gastrointestinal tract, including esophagus, duodenum, small bowel, colon, and rectum [2]. Endoscopic treatment is currently considered the first option for the management of gastrointestinal Dieulafoy lesions, whereas surgery or selective arterial embolization is advocated for cases with intractable bleeding or unsuccessful endoscopic therapy [3]. Endoscopic mechanical methods (clipping and banding) have shown good results in terms of initial and long-term hemostasis, mostly for lesions located in the upper gastrointestinal tract [4]. Hemoclips have been shown to be effective for the treatment of postpolypectomy colonic bleeding [5]. We report a case of acute hemorrhage from colonic Dieulafoy lesion that was successfully treated by endoscopic hemoclipping.

## Case Report

**Figure 1:**
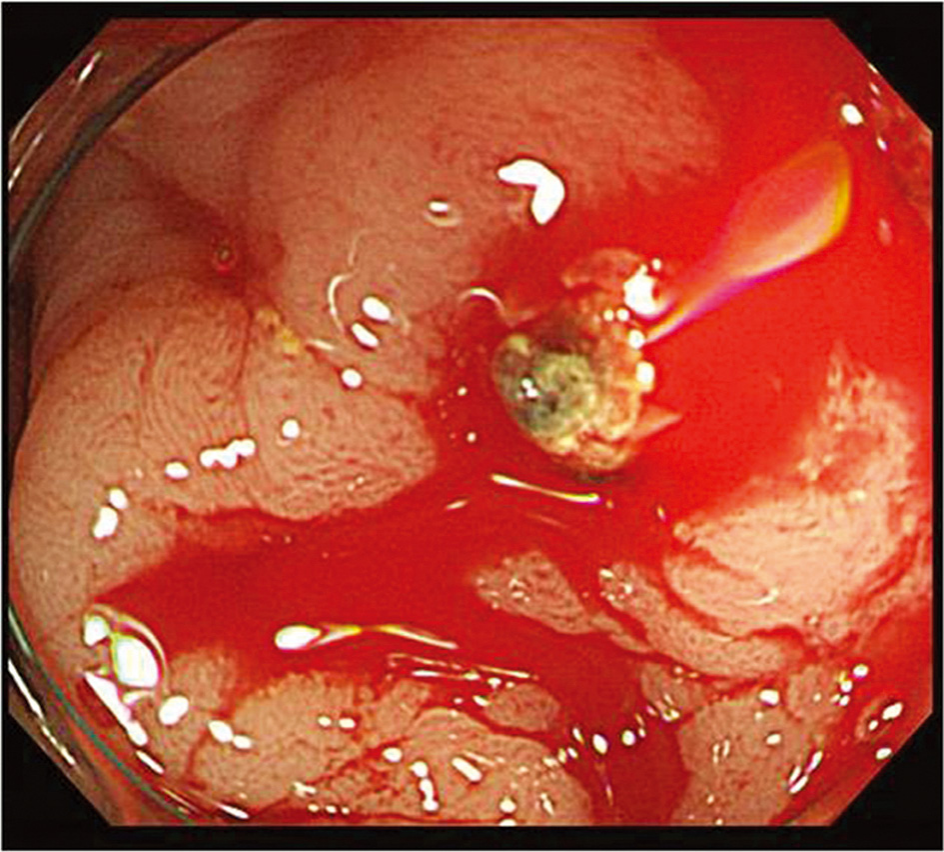
Colonoscopic examination reveals pulsatile fresh bleeding from exposed vessel without a mucosal defect or ulceration at 10 cm from proximal to anal verge.

**Figure 2:**
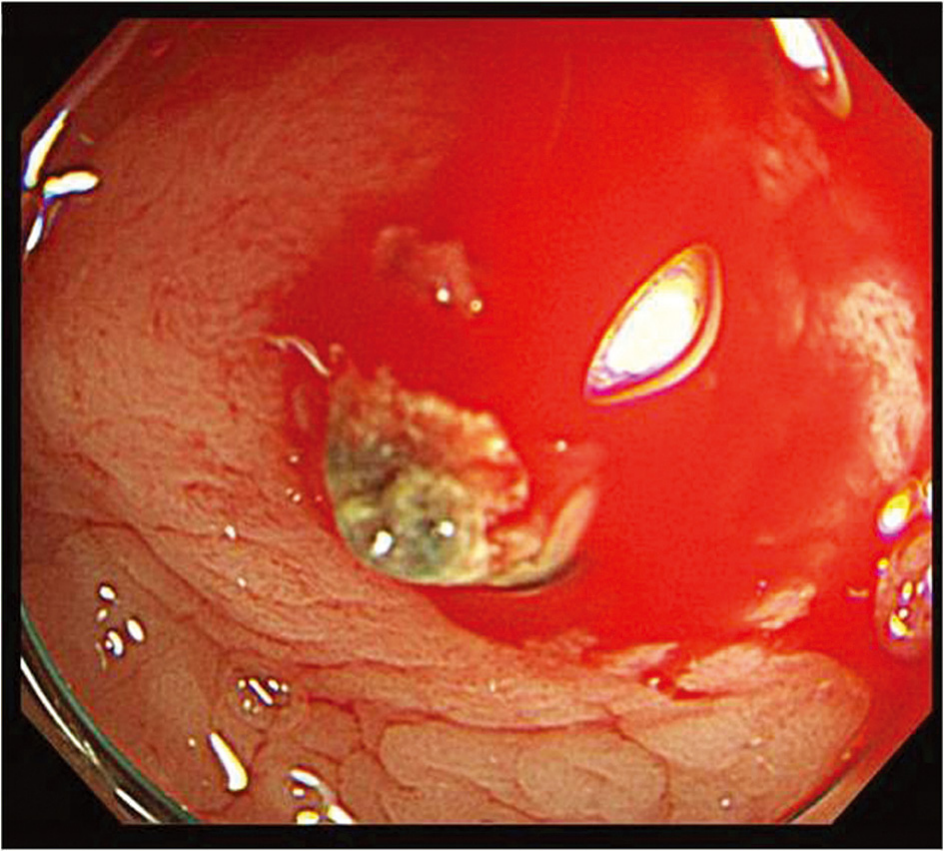
After washing with fresh water, micropulsatile streaming from a minute (< 3 mm) through normal surrounding mucosa is observed.

**Figure 3:**
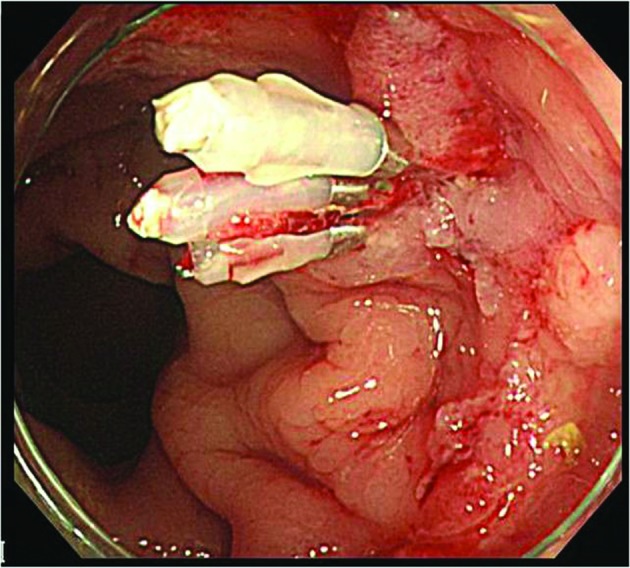
Three hemostatic clips were deployed, and the pulsatile bleeding stopped completely.

**Figure 4:**
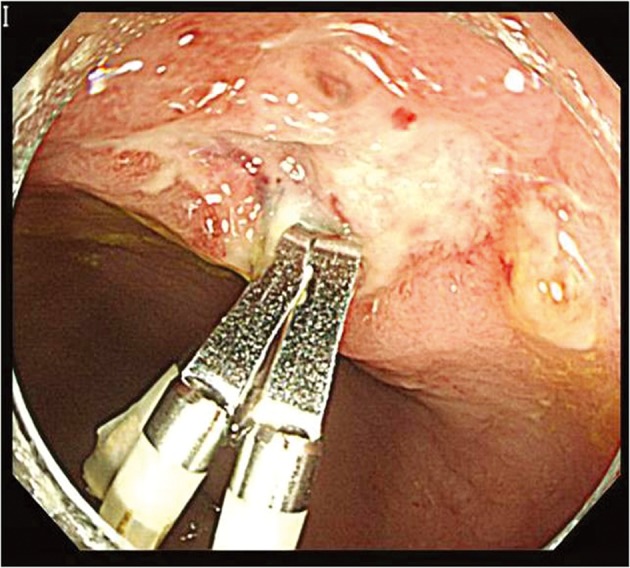
Five days after deploying hemostatic clips, colonoscopy showed well placed hemostatic clips and no evidence of bleeding and potential bleeding focus.

An 89-year-old male was admitted to our hospital with acute onset fresh rectal bleeding and hematochezia. His past medical history included diabetes mellitus and hypertension. He had no previous history of bleeding. On arrival, his pulse rate was 98 beats per minute and his systolic blood arterial pressure was 100 mmHg. The initial hematocrit was 27 percent. Physical examination revealed tenderness in the left lower quadrant without rebound tenderness. Rectal examination showed bright red blood; no masses were felt. Emergent colonoscopic examination discovered pulsatile fresh bleeding from exposed vessel without a mucosal defect or ulceration, consistent with Dieulafoy lesion, at 10 cm from proximal to anal verge (Fig. 1, 2). The lesion was It was treated with injection of 2 mL of 1/10000 epinephrine and three hemostatic clips (Fig. 3). Follow-up colonoscopy, after 5 days, showed stabilized lesion without further bleeding (Fig. 4). The patient was discharged one day later. A colonoscopy performed two months later did not demonstrate any abnormality where the hemoclip had been applied. Furthermore, the bleeding has not recurred in the six months after his discharge from hospital.

## Discussion

Dieulafoy's lesion is a well-recognized cause of gastrointestinal bleeding. The lesion is a submucosal artery that protrudes through a small mucosal defect into the lumen. Initially named exulceratio simplex by Gallard, this lesion has also been designated gastric aneurysm, submucosal arterial malformation, Dieulafoy's ulcer, cirsoid aneurysm, and caliber persistent

artery. The endoscopic criteria that define gastrointestinal Dieulafoy lesions are the following [6]: 1) active arterial spurting or micropulsatile streaming from a minute (< 3 mm) mucosal defect or through normal surrounding mucosa; 2) visualization of a protruding vessel with or without active bleeding within a minute mucosal defect or through normal surrounding mucosa; and 3) the appearance of a fresh, densely adherent clot with a narrow point of attachment to a minute mucosal defect or mucosa of normal appearance. Our first patient had a micropulsatile streaming through normal surrounding mucosa.

Surgery has been the traditional treatment of Dieulafoy lesions, but recently endoscopic therapy has become the gold standard, particularly for lesions located in the stomach, in which initial hemostasis is achieved in approximately 95 percent of cases [7]. Several endoscopic methods have been successfully used in the treatment of gastrointestinal Dieulafoy lesions. Although no single treatment modality has proven superior to any other, the only randomized trial showed a lower rebleeding rate with mechanical methods (band ligation or clip) compared with epinephrine injection [3]. To our knowledge, only six cases diagnosed with colonic Dieulafoy lesions treated with hemoclipping have been reported n English literature: one located in the cecum, one in the ascending colon, one in the transverse colon, and three in the rectum [3, 8-10]. Sone et al used hemoclipping after unsuccessful initial treatment with epinephrine [9]. The bleeding recurred because none of the clips had correctly grasped the vessel; after further hemoclips had been more accurately applied, the bleeding stopped and did not recur. Nozoe et al reported initial hemostasis with hemoclips in two patients, but follow-up was not mentioned [8]. Chung et al successfully treated one rectal lesion with hemoclips [4]. Gimeno-Garcia et al.’s approach included epinephrine injection followed by hemoclipping in both patients. We followed Gimeno-Garcia’s technique. Hemoclips can be applied without any previous treatment by injection as long as the source of bleeding can be clearly detected; however, epinephrine is considered helpful because it can reduce the amount of bleeding, enabling the prompt application of hemoclips [11].

Although the application of the hemoclip on the gastrointestinal mucosa is not considered technically demanding, it is important to apply the hemoclips accurately on Dieulafoy lesions, particularly the first one [10]. An incorrectly located hemoclip may hamper the application of subsequent hemoclips [10]. Hemoclipping is a safe and effective method for the treatment of colonic Dieulafoy lesions.
